# CXCR4^+^ Sorted Adipose-Derived Stem Cells Enhance Their Functional Benefits and Improve Cardiac Function after Myocardial Infarction

**DOI:** 10.1155/2022/6714765

**Published:** 2022-08-23

**Authors:** Zihui Yuan, Chuanqi Cai, You Qin, Kai Yan, Jian Wang

**Affiliations:** ^1^Department of Vascular Surgery, Union Hospital, Tongji Medical College, Huazhong University of Science and Technology, Wuhan 430022, China; ^2^Cancer Center, Union Hospital, Tongji Medical College, Huazhong University of Science and Technology, Wuhan 430022, China

## Abstract

**Objective:**

The homing of adipose-derived stem cells (ASCs) to infarcted myocardium, which is important for improved cardiac function, has been investigated previously, but with poor efficiency. Substantial improvements in engraftments are required to optimize ASC treatment. Stromal derived factor-1*α* (SDF-1*α*) is upregulated early after MI, and its endogenous receptor, chemokine receptor 4 (CXCR4), is pivotal in stem cell survival, migration, and engraftment. We examined whether CXCR4^+^ ASCs enhance their efficacy of migration and engraftment posttransplantation and improve heart function following myocardial infarction (MI).

**Methods and Results:**

CXCR4^+^ ASC subpopulations were sorted by fluorescence-activated cell sorting. CXCR4^+^ sorted ASCs exhibited the stronger cell viability, the faster proliferation rate, and the better migration capability in comparison with unfractionated ASCs. CXCR4^+^ sorted ASCs secreted a higher level of angiogenic growth factors including VEGF, HGF, and IGF-1 relative to unfractionated ASCs. Fewer apoptotic cells under oxygen-glucose deprivation were detected in CXCR4^+^ sorted ASCs than in unfractionated ASCs. Osteogenic and angiogenic differentiation were more pronounced in CXCR4^+^ sorted ASCs than in unfractionated ASCs. At 3 days after acute MI, rats were randomly allocated to receive intramyocardial injection of cell culture medium, CXCR4^+^ sorted ASCs, and unfractionated ASCs. Left ventricular function was assessed echocardiographically 4 weeks thereafter. Explanted hearts were then processed for the immunofluorescence detection of survived cells, quantification of angiogenesis, and cell engraftment. CXCR4^+^ sorted ASCs more obviously engrafted into infarcted myocardium, more markedly inhibited collagen remodeling, and more effectively improved heart function and promoted capillary formation than did unfractionated ASCs.

**Conclusion:**

CXCR4^+^ sorted ASCs are superior to unfractionated ASCs due to better viability, faster proliferation, more cytokine secretion, and stronger migration. CXCR4^+^ sorted ASCs provide better curative benefits on MI than do unfractionated ASCs and can be efficiently harvested and purified from adipose tissue, they may serve as a promising candidate for MI.

## 1. Introduction

A multipotential stem cell population, isolated from the stromal vascular fraction of adipose tissue digestion and elimination of mature adipocytes, is referred to as adipose-derived stem cells (ASCs) [1, 2]. ASCs are able to differentiate into endothelial cells, incorporate into newly formed blood vessels, and augment postischemic neovascularization [3–5]. ASCs have been demonstrated to secrete multiple angiogenic cytokines and antiapoptotic factors such as vascular endothelial growth factor (VEGF) and hepatocyte growth factor (HGF), at levels that are bioactive [6, 7]. Several studies investigating ASC therapy for myocardial repair have reported a modest yet significant 3% to 4% improvement in left ventricular (LV) ejection fraction [8–10].

Despite the beneficial effect of ASCs on infarcted hearts, poor cell survival in vivo and extensive cell redistribution of extracardiac organs remains the largest barriers for stem cell therapy on myocardial infarction. Transplanted cells in the ischemic tissues undergo localized hypoxia-induced oxidative stress and inflammation and are more prone to encounter apoptosis and death [11]. Most of the implanted cells died within several weeks after transplantation [12, 13]. Transplanted cells can be drained into heart veins or lymphatics and then flow to the right ventricle and lung [14, 15]. Intramyocardially injected stem cells would migrate through the collateral vessels into LV chamber, were then delivered into the peripheral circulation and retained into the spleen and liver [16, 17]. There is the possibility that promoting the survival and engraftment of transplanted stem cells might drastically improve the treatment efficacy of cell therapy.

Stromal cell-derived factor-1*α* (SDF-1*α*), a potent chemoattractant, is the most important factor that induces the migration and engraftment of stem cells into the damaged tissues [18, 19]. SDF-1*α* is significantly upregulated in the heart early after infarction [19]. C-X-C chemokine receptor type 4 (CXCR4) is the receptor for SDF-1*α*, and SDF-1*α*/CXCR4 signaling pathway plays a critical role in recruitment, homing, and subsequent engraftment of transplanted cells into the damage site [20, 21]. Of importance, CXCR4-postive cells exhibited an enhanced secretion pattern of angiogenic and antiapoptotic cytokines [22]. The migration and beneficial effects of ASCs require the cell surface expression of CXCR4. However, among the population of stem cells, only a small number expresses CXCR4 [22]. CXCR4 overexpression using a lentiviral gene transfer technique significantly increases the homing and engraftment of ASCs into ischemic hindlimb [23]. CXCR4-positive bone marrow mononuclear cells are purified by immunomagnetic bead and improve neovascularization after limb ischemia [24].

In this present study, in vitro cultured ASCs were sorted and purified using sterile fluorescence-activated cell sorting (FACS) to obtain CXCR4-postive ASCs (CXCR4^+^ sorted ASCs). To the best of our knowledge, no report has described the therapeutic effect of CXCR4^+^ sorted ASCs on myocardial infarction. We examined (1) whether CXCR4^+^ sorted ASCs exhibited greater paracrine, migration, antiapoptosis, and induction differentiation capacity in vitro and (2) whether CXCR4^+^ sorted ASCs improved their homing and engraftment in infarcted hearts and promoted cardiac functional recovery after myocardial infarction.

## 2. Materials and Methods

The animal experiments were approved by the Institutional Animal Ethical Committee (IAEC) of Huazhong University of Science and Technology, in compliance with the institutional guidelines for the care and use of animals.

### 2.1. Isolated and Culture of ASCs

Male transgenic rats expressing green fluorescent protein (GFP) were used as the cell source for all in vitro and in vivo studies. The rats were anesthetized with an intraperitoneal injection of 40 mg/kg body weight ketamine and 1 mg/kg body weight xylazine. Abdominal subcutaneous and inguinal adipose tissue are dissected, minced, and digested with 0.2% collagenase I (Worthington Biochemical Corporation, Lakewood, NJ, USA) at 37°C for 20-30 minutes. After digestion, the cell suspensions were filtered twice through a 100 *μ*m and then a 25 *μ*m nylon membrane to eliminate the undigested fragments. Cellular suspension was centrifuged at 1000 × g for 10 minutes, and supernatants were discarded. Cell pellets were resuspended in cell-culture medium (CCM) composed of high-glucose Dulbecco's modified Eagle medium (DMEM) supplemented with 10% fetal bovine serum (FBS). The cell suspensions were then seeded into culture dishes and incubated at 37°C in a humidified atmosphere containing 5% CO_2_. The CCM was changed daily to remove nonattached cells and debris. The adherent cells were referred to as ASCs.

### 2.2. CXCR4^+^ ASC Isolation and Purity Identification by FACS

Cultured ASCs were harvested by trypsinization. ASCs (1 × 10^8^ cells/ml) were resuspended in phosphate-buffered saline (PBS) contained 3% bovine serum albumin and incubated with saturating concentrations of Alex Fluor 594-conjugated rabbit anti-mouse CXCR4 antibody (Clone EPUMBR3, Abcam, Cambridge, MA, USA) for 30 minutes at 4°C. Samples were also incubated in corresponding isotype controls as Alex Fluor 594-conjugated rat IgG2a. Cellular suspension was centrifuged at 300 × g for 5 minutes. After two washes with PBS, the ASCs were resuspended in cell-sorting medium at a concentration of 1 × 10^7^ cells/ml. A fluorescence analysis cell sorter (FACS Calibur, Becton Dickinson, Mountain View, CA, USA) was utilized for sorting CXCR4^+^ purified subpopulations in channels for red fluorescence. Sample analysis rates ranged from 1000 to 3000 cells per second. The sorted fractions were immediately analyzed by the same technique for purity check. Data acquisition and analysis were then performed (Cell Quest software, Becton Dickinston).

ASCs were incubated with superparamagnetic iron oxide (SPIO) nanoparticles coated with dextrin (Feridex, Bayer Healthcare, Wayne, NJ, USA) at a concentration of 50 *μ*g/ml and protamine sulfate at a concentration of 6 *μ*g/ml for 48 hours. Efficacy of SPIO labeling was assessed by measurement of the percent of the ASCs containing SPIO particles with Prussian blue staining. At two days after incubation in SPIO-containing CCM, most of the ASCs (>90%) were stained positive for SPIO particles. Immediately prior to transplantation, the ASCs were detached with trypsin and were then centrifuged. The supernatant was discarded, and the cell pellet was resuspended in FBS-free medium.

### 2.3. MTT Assay

ASC's viability was evaluated by the 3-(4,5-methylthiazol-2-yl)-2,5-diphenyl-tetrazolium bromide (MTT) assay. Cells were plated at a density of 1 × 10^4^ cells/well in a 24-well plate and evaluated after 24 hours of culture. After washing the cells, the CCM containing 2.5 mg/ml MTT was added to each well. Cells were then incubated for 1 hour at 37°C. Formazan crystals that had formed in viable cells were solubilized with dimethyl sulfoxide. Each sample was transferred into 96-well plates, and the absorbance of each well was measured at 560 nm.

### 2.4. Cell Proliferation Assay

ASC's proliferation was evaluated by Cell Counting Kit-8 (CCK-8) assays. Briefly, ASCs were seeded in 96-well plates at a density of 1 × 10^4^ cells/well as monolayer and allowed to attach for 24 hours. After rinsing twice with PBS, a cell counting kit reagent (Sigma-Aldrich, St Louis, MO, USA) was added to each well and incubated for 2 hours at 37°C with 5% CO_2_. The optical density of the well was measured at 450 nm using a microplate reader.

### 2.5. TUNEL Assay under Oxygen Glucose Deprivation (OGD) Condition

ASCs were plated onto BSA-coated dishes and allowed to attach to cultured dishes. The next day, cells were rinsed twice with serum-free and glucose-free DMEM without sodium pyruvate and were cultured in the same medium at 37°C in an anoxia chamber saturated with gas containing 95% N_2_ and 5% CO_2_. Nonadherent and adherent cells were collected after 12 hours of OGD stimulation. Apoptotic ASCs were detected by TUNEL assay using an in situ detection kit (Roche, Mannheim, Germany). ASCs were fixed for 30 minutes in 4% paraformaldehyde, permeabilized for 10 minutes with 0.1% Triton X-100 in sodium citrate, and treated for 10 minutes with 3% H_2_O_2_. After washing three times with PBS, the cells were incubated with TUNEL reaction mixture in a humidified chamber for 60 minutes at 37°C in the dark. The cells were counter-stained with DAPI to visualize nuclei. The number of TUNEL-positive cells was counted in five randomly selected fields on a fluorescence microscope.

### 2.6. Scratch Assay

ASCs were grown in six-well plates until they reached 85%-90% confluence. At day 5 postconfluence, a sterilized 200 *μ*l disposable pipette tip was used to create a wound on the center of confluent cell monolayer. Cells were maintained in DMEM-F12 supplemented with 10% fetal bovine serum (FBS) and 1% streptomycin and penicillin. The width of the scratch between the cells bordering the wound was observed and measured by images taken under light microscopy at 0 and 24 hours. Three independent experiments were performed, and the cells in three wells of each group were quantified. Wound closure (%) indicates the percentage of wound closure with the initial scratch width set as 100%.

### 2.7. Adipogenic and Oil-Red-O Staining

For adipogenic differentiation, ASCs were incubated for 12 days in DMEM supplemented with 5% FBS, 10 *μ*M bovine insulin, 250 isobutyl-methylxanthine, 200 *μ*M indomethacin, 1 *μ*M dexamethasone, 5 *μ*g/ml streptomycin, and 5 U/ml penicillin. After 12-day adipogenic induction, the cells were fixed with 4% paraformaldehyde for 10 minutes at 37°C and then were stained with Oil Red O reagent (0.5% in isopropanol) for 15 minutes in Oil-Red-O at a ratio of 60% stock solution (0.5% weight/volume in isopropanol) for 15 minutes. Oil-Red-O stained cells were visualized by light microscopy.

### 2.8. Osteogenic Differentiation and Alkaline Phosphatase Staining

For osteogenic induction, ASCs were incubated for 28 days in DMEM supplemented with 10% FBS, 10 mM *β*-glycerophosphate, 50 *μ*M ascorbate-2-phosphate, 10 nM 1,25(OH)_2_ vitamin D_3_, 5 *μ*g/ml streptomycin, and 5 U/ml penicillin. After 28-day osteogenic induction, the cells were fixed with 4% paraformaldehyde for 10 minutes at 37°C and were then stained in alkaline phosphatase detection buffer containing nitro blue tetrazolium (NBT) and 5-Bromo-4-chloro-3-indolyl phosphate (BCIP) for 20 minutes at 37°C. Alkaline phosphatase catalyzed and hydrolyzed the BCIP to form an immediate product, which reacted with the NBT and produced an insoluble brown dye. The stained cells were then visualized under a light microscope.

### 2.9. Quantitative PCR

Total RNA was extracted from cells using TRI Reagent (Sigma-Aldrich, St Louis, MO, USA). First-strand cDNA was synthesized using 1st-strand cDNA synthesis Kit (Roche) from 1 *μ*g of total RNA. The synthesized cDNA was subjected to quantitative real-time PCR reaction with the help of StepOne™ real-time PCR system (Applied Biosystems, Foster City, CA, USA). GAPDH was used as an internal control. The gene for CXCR4 was analyzed. The genes for adipogenic differentiation were lipoprotein lipase (LPL) and peroxisome proliferator-activated receptor gamma (PPAR*γ*). The genes for osteogenic differentiation were bone sialoprotein (BSP) and osterix (OSX). The genes for angiogenic growth factors were VEGF, HGF, and IGF-1. The following primers were used: GAPDH, sense primer 5′-GCCAAGGTCATCCATGACAAC-3′ and antisense primer 5′-GTGGATGCAGGGATGATGTTC-3′, CXCR4, sense primer 5′-GGCAATGGGTTGGTAATCCT-3′ and antisense primer 5′-CGTGGACAATGGCAAGGTAG-3′, LPL, sense primer 5′-GTGAGAACATTCCCTTCACCCT-3′ and antisense primer 5′-CCAGCGGAAGTAGGAGTCGT-3′, PPAR*γ*, sense primer 5′-CCTTTACCACGGTTGATTTCTC-3′ and antisense primer 5′-CAGGCTCTACTTTGATCGCACT-3′, BSP, sense primer 5′-AGCTGACGCTGGAAAGTTGG-3′ and antisense primer 5′-TCAGTGACGCTTGCCTCCTC-3′, osterix, sense primer 5′-TCAAGCACCAATGGACTCCTC-3′ and antisense primer 5′-TATCCAAGGACGTGTAGACACTAGG-3′, VEGF, sense primer 5′-ATCTTCAAGCCGTCCTGTGTG-3′ and antisense primer 5′-AGGTTTGATCCGCATGATCTG-3′, HGF, sense primer 5′-AAGGGCTTTGATGATAATTATTGC-3′ and antisense primer 5′-TCCATTCCAAATGGTATTGGTG-3′, and IGF-1, sense primer 5′-GGCACTCTGCTTGCTCACCT-3′ and antisense primer 5′-ACTCATCCACAATGCCCGTC-3′.

### 2.10. SDF-1*α* Protein Expression in Infarcted Hearts

Three and seven days after ligation of left anterior descending (LAD) artery, the hearts of animals (*n* = 3 for each time-points) were harvested and left ventricular free wall, including infarcted myocardium, was dissected at a level just below the ligation. Three to five specimens from normal and infracted LV myocardium were subjected to immunofluorescence staining and Western blotting. Immunofluorescence staining for SDF-1*α* was performed on frozen sections. Heart tissues were embedded into OCT compound (Miles Inc., Elkhart, IN, USA). Western blot analysis was performed using lysates from myocardial tissue collection at 3 and 7 days following myocardial infarction. Proteins were separated and transferred to membranes, after which they were probed with antibody against SDF-1*α*.

### 2.11. Animal Model and Experimental Protocol

Female rats (*n* = 35) were anesthetized through inhalation of 1.5%-2% isoflurane in oxygen, followed by intubation and mechanical ventilation with a rodent ventilator at a rate of 60-70 breaths/min and tidal volume of 2 to 3 ml. An 18-gauge angiocatheter was utilized as an intubation tube throughout the procedure. The skin above the chest was shaved and sterilized. Under sterile condition, the heart was exposed through a left anterior thoracotomy at the fourth intercostal space. The left anterior descending artery (LAD) was identified after retraction of the left atrial appendage and permanently ligated at about 2-5 mm from the bottom of left atrium using a 7-0 silk suture. Myocardial infarction was confirmed by observation of a demarcation of injury with blanching of the myocardium. Sham surgery involved the same cardiac exposure without placement of the coronary suture. All rats were continuously monitored after surgery. All rats were provided an intramuscular injection of analgesics and antibiotics (penicillin, 40,000 U/0.2 ml) after surgery. Five rats were not included into the study, two sudden deaths occurred within 2-3 days after LAD ligation, two died during the recovery period following cell transplantation, and one rat suffered from incision infection.

The injection of ASCs or CCM (for the control group) was performed at three days after LAD occlusion, through the same intercostal approach for surgical exposure of the LAD region. Surviving rats received CCM (*n* = 10), unfractionated ASCs (*n* = 10), and CXCR4^+^ sorted ASCs (*n* = 10). In the rats receiving ASC implantation, GFP-positive ASCs were resuspended in 150 *μ*l of CCM (1.5 × 10^6^ cells) and intramyocardially injected at four preinfarct sites into anterior and lateral aspects of the viable myocardium bordering the infarction zone using a Hamilton syringe (Hamilton, Reno, NV, https://www.hamiltoncompany.com) with a 30-gauge beveled needle. ASCs were kept on ice prior to injection for preventing cell damage. Infarction zone manifested as the region of pale color and dysfunction downstream from the ligature. The infarcted and boarding regions were carefully dissected away from the normal myocardium. Rats (*n* = 5 for each cell transplanted group) underwent the intramyocardial injection of SPIO-labeled ASCs. CCM control rats underwent four injections of 150 *μ*l CCM in the same regions. After cell transplantation, the chest wall, muscle layers, and skin were closed with a 3-0 silk sutures. Four weeks posttransplantation, echocardiography was performed under mild isoflurane sedation. After echocardiographic examination, rats were sacrificed, and the hearts were removed and frozen down in liquid nitrogen until further processing.

### 2.12. Hematoxylin-Eosin (HE), Prussian Blue, and Masson's Trichrome Staining

The frozen rat hearts were transversely cryosectioned into 8 *μ*m thick slices from apex to the level just below the coronary artery ligation site. Heart tissue sections were immersed in Prussian blue reagent (4% potassium ferrocyanide/12% HCl, 50 : 50 vol/vol) for 40 minutes under agitation. The tissue sections were then washed once in PBS and twice in deionized water. The tissue sections were counterstained through standard HE procedure. Then, the slides were mounted by antifade mounting media. Alternating tissue sections were stained with Masson's trichrome to delineate the infarct region. The sections were examined under a light microscope.

### 2.13. Immunofluorescence Analysis

Adherent cells, grown in chamber slides, were washed with PBS and fixed overnight at 4°C in 3.7% paraformaldehyde/PBS buffer. After blocking for 1 hour in 1% BSA PBS buffer, cells were incubated for 1 hour in 0.3% Triton X-100/PBS buffer with primary antibody against SDF-1*α* (Abcam, Cambridge, MA, USA). After washing, a second antibody, Alexa Fluor 594-conjugated anti-rabbit IgG (Invitrogen, Carlsbad, CA, USA) was applied for an additional 60 minutes at room temperature.

Tissue sections were fixed in cold methanol for 10 minutes, incubated in PBS with 0.1% Triton X-100 for 15 minutes, and then blocked in 2% goat serum and 1% bovine serum albumin (BSA) for 1 hour at 37°C. Slides were then incubated with primary antibodies at 4°C overnight. SDF-1*α*, a potent ligand for CXCR4, was identified by rabbit anti-SDF-1*α* antibody (Abcam, Cambridge, MA, USA). A rabbit anti-CXCR4 antibody (Abcam, Cambridge, MA, USA) was used to label the grafted CXCR4-positive cells. von Willebrand factor (vWF), an endothelial cell marker, was detected by rabbit monoclonal anti-vWF antibody (Abcam, Cambridge, MA, USA). After being washed three times with PBS, slides were incubated with Alexa Fluor 594-conjugated goat anti-rabbit (Invitrogen, Carlsbad, CA, USA) second antibody at room temperature for 1 hour. After three more washes with PBS, the sections were mounted in mounting reagent (Santa Cruz Biotechnology, Dallas, TX, USA) with 4′,6-diamidino-2-phenylindole (DAPI) (Sigma-Aldrich Corp., St Louis, MO, USA) and visualized on the AxioScope epifluorescence microscopy (Carl Zeiss, Oberkochen, Germany).

### 2.14. Western Blot Analysis

Collected cells were solubilized in ice-cold RIPA lysis buffer. Myocardial tissue from the infarcted hearts was removed and homogenized in ice-cold RIPA lysis buffer. Lysis buffer was supplemented with protease inhibitors. The lysate was centrifuged at 12,000 × g for 5 minutes. The supernatant was collected after centrifugation. Protein concentration was determined using the Bradford protein assay.

Equal amounts of total protein were separated on 10%-12% glycine gel and transferred onto polyvinylidene difluoride membranes. The membranes were blocked in 5% nonfat dry milk in PBS with Tween-20 and incubated with primary rabbit anti SDF-1*α* monoclonal antibody or rabbit anti CXCR4 monoclonal antibody (Abcam, Cambridge, MA, USA) at dilution of 1 : 1000 overnight at 4°C. Glyceraldehyde-3-phosphate dehydrogenase (GAPDH) expression served as an internal control. After being washed in PBS, the membranes were incubated with horseradish peroxidase- (HRP-) conjugated anti-rabbit secondary antibodies for 1 hour at room temperature. Protein bands were visualized by enhanced chemiluminescence (ECL) kit before exposure to X-ray film. Target protein band intensity was normalized by GAPDH and quantified by the Image J software.

### 2.15. Two-Dimensional Transthoracic Echocardiography

Heart function was assessed by transthoracic echocardiography 4 weeks after MI using a Philips Sonos 5500 ultrasound diagnostic system (USA) equipped with an S4 probe whose frequency is 2.5 to 4.2 MHz. After anesthetization by inhalation of 2.5% isoflurane, rats were placed in the left lateral decubitus position. Two-dimensional (2D) mode image of parasternal short-axis view was acquired to measure left atrial diameter and then shifted to position the motion mode (M-mode) cursor at the level of the papillary muscles and perpendicular to the interventricular septum and LV free wall. LV end-diastolic diameter (LVEDD) and LV end-systolic diameter (LVESD) were obtained from two-dimensional images together with M-mode interrogation in long-axis view. LV fractional shortening (FS) was calculated as an index of systolic function: LVFS (%) = (LVEDD–LVESD)/LVEDD × 100. LV ejection fraction (LVEF) was determined by modified single-plane Simpson's rule in the apical four-chamber oblique view. All echocardiographic measurements were averaged from at least three separate cardiac cycles.

### 2.16. Statistical Analysis

All statistical analyses were performed using the SPSS software (IBM Corp., Armonk, NY, USA). Continuous variables were expressed as the mean ± standard deviation with normal distribution. Unpaired Student's *t*-test was used to make comparisons between two groups. When three or more groups were compared, multiple comparisons were made using two-way analysis of variance (ANOVA). Values of *p* < 0.05 was considered statistically significant. All experiments were performed in triplicate.

## 3. Results

### 3.1. The Expression of SDF-1*α* Was Upregulated in the Infarcted Myocardium

**Figure 1:**
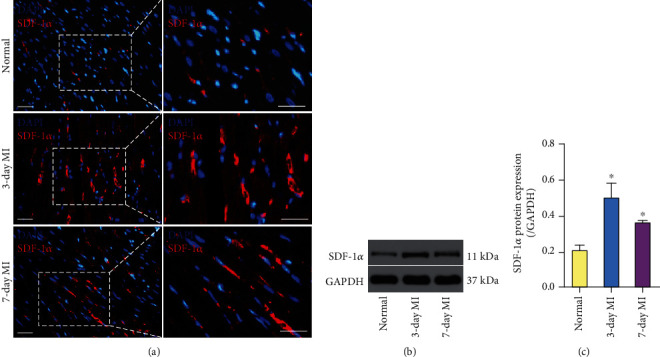
SDF-1*α* was upregulated in infarcted myocardium. (a) SDF-1*α* (red) was substantially observed on the vessel wall in infarcted myocardium at 3 and 7 days after myocardial infarction, which was scantly expressed in remote normal myocardium. (b, c) The protein expression of SDF-1*α* in infarcted myocardium peaked at 3 days after myocardial infarction and remained elevated at 7 days after myocardial infarction relative to remote normal myocardium. Scale bar represents 50 *μ*m. ^∗^*p* < .05 versus remote normal myocardium. Abbreviations: SDF-1*α*: stromal cell-derived factor-1*α*; MI: myocardial infarction; GAPDH: glyceraldehyde-3-phosphate dehydrogenase.

Immunofluorescence staining showed that interstitial tissue and blood vessels of infarcted myocardium highly expressed SDF-1*α* at 3 and 7 days following myocardial infarction in contrast to the remote normal myocardium (Figure 1(a)). The protein expression of SDF-1*α* in infarcted myocardium peaked at 3 days and remained elevated at 7 days over baseline values in remote normal myocardium (Figures 1(b) and 1(c)).

### 3.2. CXCR4^+^ Subpopulations Were Sorted from ASCs by FACS

**Figure 2:**
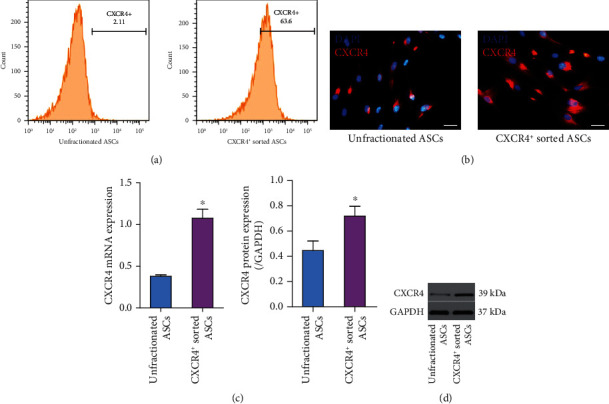
CXCR4^+^ ASCs were isolated and sorted by fluorescence-activated cell sorting (FACS). (a) Only a minor population of CXCR4^+^ ASCs (≈2%) was detected in unfractionated ASCs. After sorting, CXCR4^+^ ASCs were enriched to approximately 63%. (b) CXCR4 (red) was highly expressed in CXCR4^+^ sorted ASCs by immunofluorescence staining. (c) The mRNA expression of CXCR4 was significantly augmented in CXCR4^+^ sorted ASCs relative to unfractionated ASCs by quantitative PCR. (d) CXCR4 protein (39 kDa) was more strongly expressed in CXCR4^+^ sorted ASCs than in unfractionated ASCs. Scale bar represents 50 *μ*m. ^∗^*p* < .05 versus unfractionated ASCs. Abbreviations: CXCR4: CXC-chemokine receptor 4; ASCs: adipose-derived stem cells; FACS: fluorescence-activated cell sorting; GAPDH: glyceraldehyde-3-phosphate dehydrogenase, DAPI, RNA, deoxyribonucleic acid; DAPI; 4′,6-diamidino-2-phenylindole.

Unfractionated ASCs were stained for PE-conjugated anti-CXCR4 antibody and sorted by FACS. The initial proportion of CXCR4^+^ populations in presorted ASCs was about 2%, and CXCR4^+^ fractions were concentrated to approximately 64% after sorting (Figure 2(a)). Immunofluorescence observation revealed a significantly stronger expression of CXCR4 in CXCR4^+^ sorted ASCs than in unfractionated ASCs (Figure 2(b)). The mRNA expression of CXCR4 was more increased in CXCR4^+^ sorted ASCs than in unfractionated ASCs (Figure 2(c)). The protein expression of CXCR4 was significantly higher in CXCR4^+^ sorted ASCs than in unfractionated ASCs (Figure 2(d)).

### 3.3. CXCR4^+^ Sorted ASCs Exhibited the Enhanced Cell Viability, Proliferation, Paracrine Actions, and Migration In Vitro

**Figure 3:**
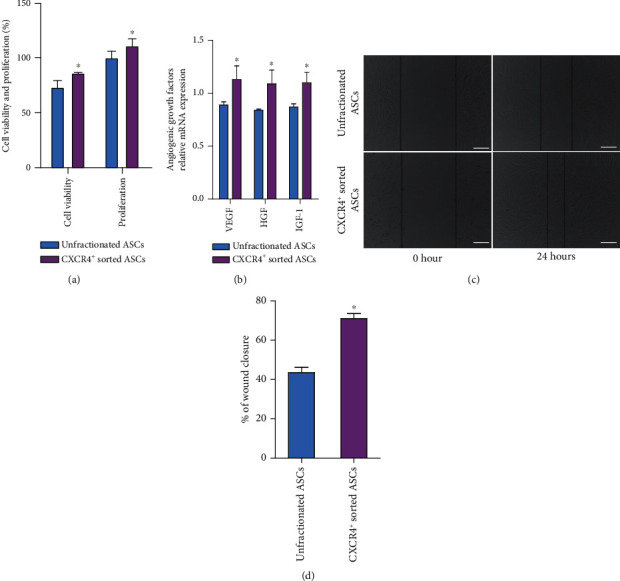
CXCR4^+^ sorted ASCs exhibited the better viability, proliferation, cytokine secretion, and migration than did unfractionated ASCs in vitro. (a) CXCR4^+^ sorted ASCs had markedly increased cell viability and proliferation rates compared with unfractionated ASCs. (b) CXCR4^+^ sorted ASCs secreted a higher level of angiogenic growth factors including VGEF, HGF, and IGF-1 relative to unfractionated ASCs. (c) Images were taken at 0 hour and 24 hours after scratch to visualize migrated cells and wound healing. (d) Scratch assay found a significantly higher wound closure percentage in CXCR4^+^ sorted ASCs than in unfractionated ASCs. Scale bar represents 200 *μ*m. ^∗^*p* < .05 versus unfractionated ASCs. Abbreviations: CXCR4: CXC-chemokine receptor 4; ASCs: adipose-derived stem cells; VEGF: vascular endothelial growth factor; HGF: hepatocyte growth factor; IGF-1: insulin-like growth factor.

Cell viability and proliferation rates were found to be higher in CXCR4^+^ sorted ASCs than in unfractionated ASCs (Figure 3(a)). The mRNA expression of angiogenic growth factors, including VEGF, HGF, and IGF-1, was markedly increased in CXCR4^+^ sorted ASCs than in unfractionated ASCs (Figure 3(b)). To evaluate the migration ability of ASCs, the wound healing was analyzed. The similar distance in two cell groups was made in the center of cell monolayers at 0 h. CXCR4^+^ sorted ASCs migrated a longer distance (migrated above 70% of the scratch) than unfractionated ASCs at 24 h (Figures 3(c) and 3(d)).

### 3.4. CXCR4^+^ Sorted ASCs Displayed More Robust Induction Differentiation and Antiapoptosis Capacity

**Figure 4:**
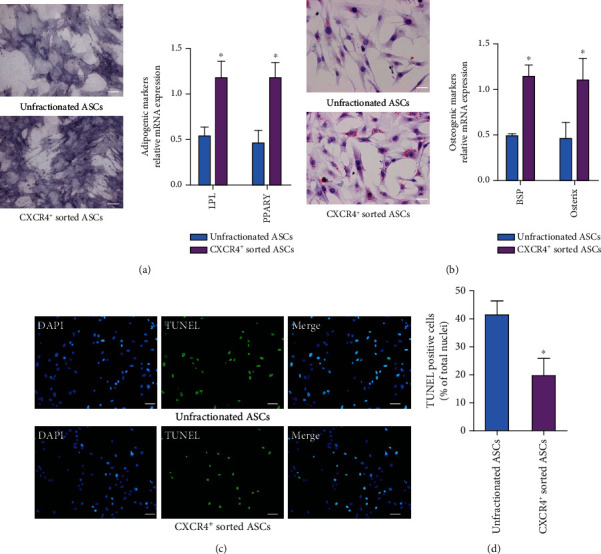
CXCR4^+^ sorted ASCs expressed higher levels of osteogenic and adipogenic marker mRNA and had lower percentage of OGD-induced apoptosis in vitro. (a) Osteocyte lineage differentiation potential was demonstrated by AP staining. CXCR4^+^ sorted ASCs exhibited the markedly increased mRNA expression of BSP and osterix in comparison with unfractionated ASCs. (b) Oil Red O staining indicated the accumulation of oil droplets in cells that differentiated to adipocytes. LPL and PPAR*γ* mRNA expressions were more detectable in CXCR4^+^ sorted ASCs as compared to unfractionated ASCs. (c) Apoptotic cell death was assessed by TUNEL staining (red fluorescence), and cell nuclei were visualized with DAPI staining (blue). OGD-induced cell apoptosis was more frequent in CXCR4^+^ sorted ASCs than in unfractionated ASCs. Scale bar represents 50 *μ*m. ^∗^*p* < .05 versus unfractionated ASCs. Abbreviations: CXCR4: CXC-chemokine receptor 4; ASCs: adipose-derived stem cells; OGD: oxygen and glucose deprivation; AP: alkaline phosphatase; BSP: bone sialoprotein; LPL: lipoprotein lipase; PPAR*γ*: peroxisome proliferator-activated receptor gamma.

In vitro test using the appropriate inductive culture conditions promoted osteogenic and adipogenic ASC differentiation (Figures 4(a) and 4(b)). The mRNA levels of osteogenic markers (BSP and osterix) were higher in CXCR4^+^ sorted ASCs than in unfractionated ASCs (Figure 4(a)). The mRNA expressions of angiogenic markers (LPL and PPAR*γ*) were increased significantly in CXCR4^+^ sorted ASCs compared to unfractionated ASCs (Figure 4(b)). Apoptosis in ASCs was initiated by 12-hour OGD. The percentage of apoptotic cells in CXCR4^+^ sorted ASCs was significantly reduced after OGD treatment compared to unfractionated ASCs (Figures 4(c) and 4(d)).

### 3.5. CXCR4^+^ Sorted ASCs Engrafted into Infarcted Myocardium More Robustly than Did Unfractionated ASCs

**Figure 5:**
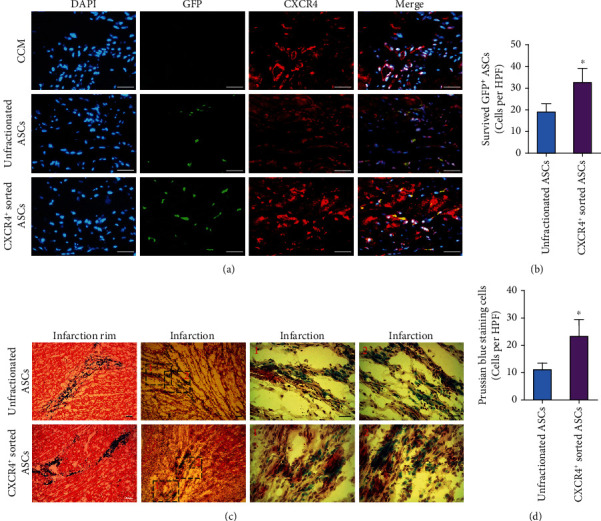
CXCR4^+^ sorted ASCs exhibited more robust engraftment into infracted myocardium than did unfractionated ASCs in vivo. (a) CXCR4^+^ and GFP^+^ ASCs were detected at 4 weeks posttransplantation in CXCR4^+^ sorted ASC-treated rats. (b) CXCR4^+^ sorted ASCs showed much higher engraftment than unfractionated ASCs. (c) SPIO-labeled ASCs were transplanted into viable myocardium bordering infarct zone and engrafted into infarcted myocardium with duration of recovery. (d) More cells containing blue-stained particles were detected in CXCR4^+^ sorted ASC-treated rats than in unfractionated ASC-treated rats. Scale bar represents 50 *μ*m. ^∗^*p* < .05 versus unfractionated ASCs. Abbreviations: CXCR4: CXC-chemokine receptor 4; ASCs: adipose-derived stem cells; GFP: green fluorescent protein; DAPI: 4′,6-diamidino-2-phenylindole.

Homing and engraftment of transplanted cells were assessed at four weeks posttransplantation. ASCs were isolated from GFP transgenic rats. Transplanted CXCR4^+^ ASCs were detected by immunofluorescence staining. GFP and CXCR4 were colocalized and coexpressed in infarcted hearts receiving CXCR4^+^ sorted ASC transplantation (Figure 5(a)). GFP^+^ and CXCR4^+^ ASCs survived, migrated, and engrafted to a higher degree into damaged myocardium in comparison with unfractionated ASCs expressing GFP alone (Figure 5(b)).

Prussian blue staining might reflect the location of SPIO-labeled ASCs engrafted in infarcted myocardium. SPIO-labeled ASCs were implanted into viable myocardium in infarct rim and migrated into the infarcted scar (Figure 5(c)). More cells containing blue-stained particles were distributed in CXCR4^+^ sorted ASC-treated rats than in unfractionated ASC-treated rats (Figure 5(d)).

### 3.6. CXCR4^+^ Sorted ASCs Increased Capillary Density More Effectively than Did Unfractionated ASCs

**Figure 6:**
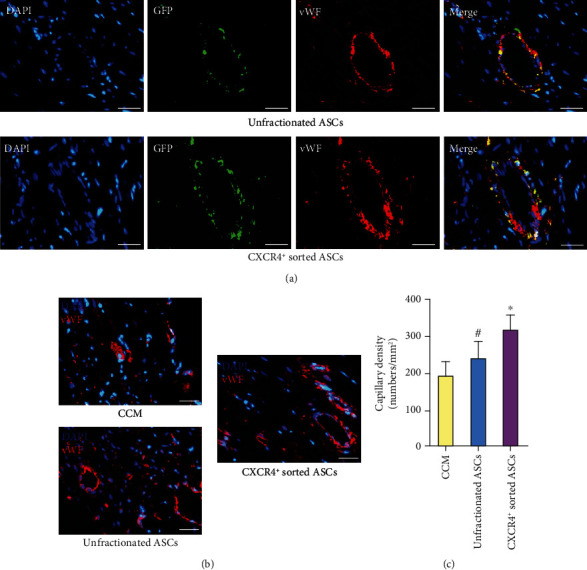
CXCR4^+^ sorted ASCs more effectively improved capillary formation than did unfractionated ASCs in vivo. (a) Some implanted GFP^+^ ASCs directly incorporated into capillary vasculature. (b) vWF staining positivity was present in the endothelial cells from capillaries. (c) Capillary density was significantly higher in CXCR4^+^ sorted ASC-treated rats than in unfractionated ASCs or CCM-treated rats. Scale bar represents 50 *μ*m. ^#^*p* < .05 versus CCM. ^∗^*p* < .05 versus unfractionated ASCs. Abbreviations: CXCR4: CXC-chemokine receptor 4; ASCs: adipose-derived stem cells; GFP: green fluorescent protein; DAPI: 4′,6-diamidino-2-phenylindole; vWF: von Willebrand factor; CCM: cell culture medium.

Capillaries were identified according to positive vWF staining in the peri-infarct zone four weeks after cell transplantation. The GFP-positive ASCs homed to the infarcted myocardium and incorporated into capillary vasculature four weeks after cell administration (Figure 6(a)). Capillary density was substantially higher in CXCR4^+^ sorted ASC-treated rats than in unfractionated ASC-treated rats and in CCM control rats (Figures 6(b) and 6(c)).

### 3.7. CXCR4^+^ Sorted ASCs Offered Better Cardiac Functional Recovery and Inhibition of Cardiac Fibrosis than Did Unfractionated ASCs

**Figure 7:**
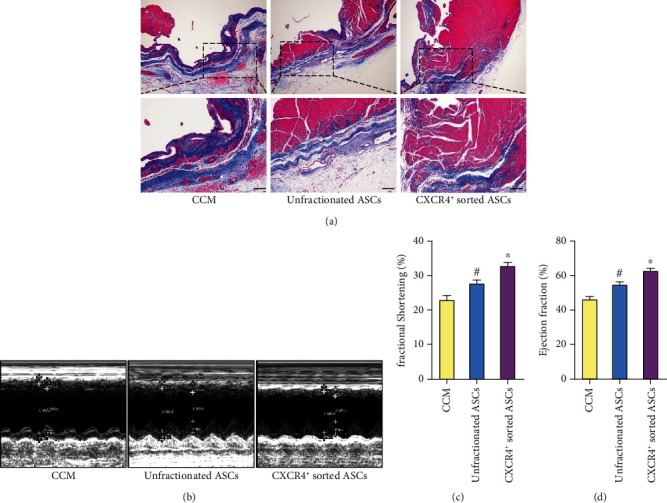
CXCR4^+^ sorted ASCs more potently improved cardiac contractility and inhibited scar formation than did unfractionated ASCs in vivo. (a) Representative Masson's trichrome staining was depicted (blue, fibrous tissue and red, viable myocardium). More viable myocardium and less fibrous tissue were observed in CXCR4^+^ sorted ASC-treated rats than in unfractionated ASC-treated rats and in CCM control rats. (b) Representative M-mode echocardiographic data in infarcted hearts receiving CCM, unfractionated ASCs, and CXCR4^+^ sorted ASCs at 4 weeks posttransplantation. (c) Comparison of ejection fraction and fractional shortening as assessed by echocardiography. CXCR4^+^ sorted ASC-treated rats demonstrated a statistically significant improvement in ejection fraction and fractional shortening at 4 weeks posttransplantation as compared with CCM or unfractionated ASC-treated rats. Scale bar represents 50 *μ*m. ^#^*p* < .05 versus CCM. ^∗^*p* < .05 versus unfractionated ASCs. Abbreviations: CXCR4: CXC-chemokine receptor 4; ASCs: adipose-derived stem cells; CCM: cell culture medium.

Masson's trichrome showed more viable myocardium and less fibrous tissue in CXCR4^+^ sorted ASC-treated rats than in unfractionated ASC-treated rats and in CCM control rats (Figure 7(a)). To determine the effect of ASC transplant on myocardial left ventricular function, we carried out echocardiographic assessments at four weeks posttransplantation.  Figure 7(b) shows three representative echocardiograms of hearts receiving CCM, unfractionated ASCs, and CXCR4^+^ sorted ASCs at four weeks after cell transplantation. LV ejection fraction and fractional shortening were significantly higher in CXCR4^+^ sorted ASC-treated rats than in unfractionated ASC-treated rats and in CCM control rats (Figures 7(c) and 7(d)).

## 4. Discussion

CXCR4 is present as a nanocluster in the plasma membrane, comprising a M-terminal extracellular domain and a C-terminal intracellular domain. The typical ligand of CXCR4 is SDF-1*α*. The interaction of CXCR4 with SDF-1*α* activates G protein subunits and the downstream Ca^2+^ mobilization from intracellular stores as well as P13K/Akt signaling pathways [25]. Activation of P13K/Akt is involved in the promotion of cell migration, proliferation, and survival [25]. Signal pathway downstream CXCR4 motivates cell migration and cell proliferation, which are also dependent on mTORC signaling. mTORC activation reinforces the anabolic metabolism that is necessary for cell growth. Hypoxia pretreatment of stem cell enhances the ability of proliferation, migration, and cell fusion by inducing CXCR4 signaling [26].

CXCR4/SDF-1*α* axis is required for survival, proliferation, and paracrine secretion of stem cells in vivo following the transplantation [27]. We compared the survival, proliferation, and cytokine secretion of unfractionated ASCs and CXCR4^+^ ASCs sorted by FACS. Compared with unfractionated ASCs, CXCR4^+^ sorted ASCs had greater survival, proliferation, and paracrine capacity in vitro. These are consistent with previous publications. CXCR knockout donor bone marrow stromal cells (BMSCs) exhibited markedly less survival and proliferation potential than do wild-type donor BMSCs after transplantation into cerebral infarct [27]. Knockout of CXCR4 significantly impaired the viability and depressed the proliferation in mesenchymal stem cells (MSCs), whereas upregulation of CXCR4 by lentiviral transfection enhances MSC viability and proliferation [28]. Isolated CXCR4^+^ BMSCs by magnetic beads secreted higher levels of proangiogenic growth factors compared to CXCR4^−^ BMSCs [24]. MSCs expressing genetically modified CXCR4 released more angiogenic growth factors such as VEGF and HGF [28].

One of the main concerns is whether or not the antibody for FACS does hamper the functionality of CXCR4 and CXCR4/SDF-1*α* binding. We compared the migration capacity of freshly isolated ASCs and CXCR4^+^ ASCs sorted with Alex Fluor 594-conjugated rabbit anti-mouse CXCR4 antibody. CXCR4^+^ sorted ASCs possessed more robust migration capacity in vitro under the stimulation of SDF-1*α*. The two previous studies have expressed the similar concerns on whether antibody for sorting hinders CXCR4 activity. CXCR4 antibody pretreatment did not change SDF-1*α*-induced invasion capacity of BMSCs [24]. Migration did not differ between freshly isolated BMSCs and BMSCs preincubated with CXCR4 antibody [29]. Taken together, these selected CXCR4 antibody recognizes were not the binding domains of CXCR4, and these antibodies can be utilized in FACS sorting for CXCR4^+^ cells [24, 29].

Stem cell therapy was limited by low cellular survival rate after transplantation due to cell apoptosis. We comparatively investigated the proportion of apoptotic cells between CXCR4^+^ sorted ASCs and unfractionated ASCs under OGD. OGD-induced apoptosis occurred more frequently in the unfractionated ASCs than in the CXCR4^+^ sorted ASCs. ASCs have multilineage potential as these cells can be induced to differentiate into adipogenic and osteogenic lineages in vitro [30–32]. Interestingly, we found that adipogenic and osteogenic marker mRNA levels were significantly higher in the CXCR4^+^ sorted ASCs than in the unfractionated ASCs. These data demonstrate that pluripotent ASCs are enriched in the CXCR4^+^ subpopulation than in the unfractionated subpopulation.

Our findings indicated that CXCR4^+^ sorted ASCs represented a subpopulation capable of adhering to endothelial cells and migrating into infracted tissue more efficiently than did unfractionated ASCs, eventually improving cardiac function after myocardial infarction. The chemokine SDF-1*α*, upregulated early after myocardial infarction, attracts the homing and engraftment of CXCR4^+^ sorted ASCs to target region via SDF-1*α*/CXCR4 interaction. The beneficial effects of CXCR4 on cell proliferation and survival are extremely vital in a therapeutic context. The longer that transplanted CXCR4^+^ sorted ASCs retain their specific characteristics, the more transplanted ASCs can cycle and accumulate in ischemic regions, and the more cytokines and growth factors can be produced from ASCs, facilitating the inhibition of cardiomyocyte apoptosis and ultimately participating in the repair of damaged heart. These results are in agreement with three previous studies. Administration of CXCR4^+^ bone marrow mononuclear cells (BMMNCs) sorted by magnetic beads significantly enhanced the perfusion recovery in mice after hindlimb ischemia compared to CXCR4^−^ BMMNCs [24]. CXCR4^+^ BMMNCs could be easily and efficiently harvested and purified from unfractionated mouse BMMNCs, exhibited more robust migration into ischemic brain tissue and more effectively reduced infarction volume and neurologic deficits than did unfractionated BMMNCs [29]. CXCR4^+^ hematopoietic stem cells (HSCs) were sorted by cytofluorometry using monoclonal anti-CXCR4 antibodies and improved LV ejection fraction following local injection in patients with chronic myocardial infarction [33].

Angiogenic growth factors including VEGF, HGF, and IGF-1 are uniquely enriched in CXCR4^+^ASCs. Our data suggest that CXCR4^+^ASCs possess the increased capacity of antiapoptosis, migration, and differentiation. Biological function of CXCR4+ ASCs are quite similar to human induced pluripotent stem cell-derived MSCs (iPSC-derived MSCs). The greater curative potential of iPSC-derived MSCs may be attributable to their superior antiapoptotic capacity [34], potent paracrine cytokines [35], and strong survival and engraftment [36] after transplantation to enhance their therapeutic efficacy.

## 5. Limitations

Several limitations to this study need to be acknowledged. First, the sample size is relatively small, but acceptable as this is an exploratory study to reveal the principle for CXCR4^+^ sorted ASC therapy. Second, we failed to investigate the ability of CXCR4^+^ sorted ASCs to achieve long-term graft survival in an animal model of myocardial infarction. In out next study, we will examine whether the CXCR4 can also improve the long-term (6 months) residency of ASCs in infarcted hearts. Third, the recent study is largely an observation examination, and the detailed molecular mechanism by which CXCR4 enhanced the survival, migration, and engraftment of transplanted ASCs is not investigated in-depth. Fourth, SDF-1*α* is significantly upregulated in infarcted myocardium and plays a key role in the homing of CXCR4^+^ASCs to ischemic heart. However, this study did not provide the direct evidence on coupling between SDF-1*α* and CXCR4.

## 6. Conclusions

SDF-1*α*, a specific ligand of CXCR4, is significantly upregulated in the interstitial tissues and blood vessels of infarcted myocardium at three and seven days postinfarction. ASCs are safely and effectively sorted and purified to harvest CXCR4^+^ ASCs using sterile FACS. CXCR4^+^ sorted ASCs were enriched to approximately 65%, and only a minor fraction of CXCR4^+^ ASCs (≈2%) was detected in unfractionated ASCs. CXCR4+ sorted ASCs were superior to unfractionated ASCs in surviving, proliferating, and induction differentiation in vitro and secrete a higher level of angiogenic growth factors. CXCR4^+^ sorted ASCs exhibit more robust migration and engraftment into infarcted myocardium and provide better cardiac functional recovery after myocardial infarction than do unfractionated ASCs; they may serve as a promising candidate for myocardial infarction.

## Data Availability

The raw data supporting the conclusions of this article will be made available by the authors, without undue reservation.
